# Contextualizing the impact of prenatal alcohol and tobacco exposure on neurodevelopment in a South African birth cohort: an analysis from the socioecological perspective

**DOI:** 10.3389/fnint.2023.1104788

**Published:** 2023-07-18

**Authors:** Yingjing Xia, Vida Rebello, Stefanie C. Bodison, Deborah Jonker, Babette Steigelmann, Kirsten A. Donald, Weslin Charles, Dan J. Stein, Jonathan Ipser, Hedyeh Ahmadi, Eric Kan, Elizabeth R. Sowell, Katherine L. Narr, Shantanu H. Joshi, Hein J. Odendaal, Kristina A. Uban

**Affiliations:** ^1^Public Health, University of California, Irvine, Irvine, CA, United States; ^2^Department of Occupational Therapy, College of Public Health and Health Professions, University of Florida, Gainesville, FL, United States; ^3^Department of Paediatrics and Child Health, University of Cape Town, Cape Town, South Africa; ^4^Department of Psychiatry and Mental Health, University of Cape Town, Cape Town, South Africa; ^5^Neuroscience Institute, University of Cape Town, Cape Town, South Africa; ^6^South African Medical Research Council (SAMRC), Unit on Risk and Resilience in Mental Disorders, Cape Town, South Africa; ^7^University Statistical Consulting, LLC, Irvine, CA, United States; ^8^Department of Pediatrics, Keck School of Medicine, Children’s Hospital Los Angeles, University of Southern California, Los Angeles, CA, United States; ^9^Department of Psychiatry and Biobehavioral Sciences, University of California, Los Angeles, Los Angeles, CA, United States; ^10^Ahmanson-Lovelace Brain Mapping Center, Department of Neurology, Geffen School of Medicine, University of California, Los Angeles, Los Angeles, CA, United States; ^11^Department of Bioengineering, University of California, Los Angeles, Los Angeles, CA, United States; ^12^Department of Obstetrics and Gynaecology, Stellenbosch University, Cape Town, South Africa

**Keywords:** socioeconomic resources, prenatal substance exposure, neurodevelopment, adverse childhood experiences, prenatal alcohol exposure, prenatal tobacco exposure

## Abstract

**Background:**

Alcohol and tobacco are known teratogens. Historically, more severe prenatal alcohol exposure (PAE) and prenatal tobacco exposure (PTE) have been examined as the principal predictor of neurodevelopmental alterations, with little incorporation of lower doses or ecological contextual factors that can also impact neurodevelopment, such as socioeconomic resources (SER) or adverse childhood experiences (ACEs). Here, a novel analytical approach informed by a socio-ecological perspective was used to examine the associations between SER, PAE and/or PTE, and ACEs, and their effects on neurodevelopment.

**Methods:**

*N* = 313 mother-child dyads were recruited from a prospective birth cohort with maternal report of PAE and PTE, and cross-sectional structural brain neuroimaging of child acquired via 3T scanner at ages 8–11 years. *In utero* SER was measured by maternal education, household income, and home utility availability. The child’s ACEs were measured by self-report assisted by the researcher. PAE was grouped into early exposure (<12 weeks), continued exposure (>=12 weeks), and no exposure controls. PTE was grouped into exposed and non-exposed controls.

**Results:**

Greater access to SER during pregnancy was associated with fewer ACEs (maternal education: β = −0.293,*p* = 0.01; phone access: β = −0.968,*p* = 0.05). PTE partially mediated the association between SER and ACEs, where greater SER reduced the likelihood of PTE, which was positively associated with ACEs (β = 1.110,*p* = 0.01). SER was associated with alterations in superior frontal (β = −1336.036, *q* = 0.046), lateral orbitofrontal (β = −513.865, *q* = 0.046), caudal anterior cingulate volumes (β = −222.982, *q* = 0.046), with access to phone negatively associated with all three brain volumes. Access to water was positively associated with superior frontal volume (β=1569.527, *q* = 0.013). PTE was associated with smaller volumes of lateral orbitofrontal (β = −331.000, *q* = 0.033) and nucleus accumbens regions (β = −34.800, *q* = 0.033).

**Conclusion:**

Research on neurodevelopment following community-levels of PAE and PTE should more regularly consider the ecological context to accelerate understanding of teratogenic outcomes. Further research is needed to replicate this novel conceptual approach with varying PAE and PTE patterns, to disentangle the interplay between dose, community-level and individual-level risk factors on neurodevelopment.

## 1. Introduction

Alcohol and tobacco are established teratogens, as proven in animal models, and consistent with findings in human pediatric samples. Numerous studies have shown that prenatal alcohol exposure (PAE) can lead to alterations in children’s physical, cognitive, mental, behavioral and neural development (Glass et al., 2014; Mattson et al., 2019). Since the original recognition of alcohol as a teratogen in humans (Jones and Smith, 1973), the subsequent 50 years of original brain research on FASD has consistently demonstrated structural brain alterations (Riley et al., 1995; Mattson et al., 1996; Archibald et al., 2001; Sowell et al., 2001, 2002, 2008). PAE poses cumulative harm to global health and results in significant economic burdens. PAE can increase demands on health care, special education, justice system, morbidity and mortality, and loss in productivity for both the affected children and their caregivers (Greenmyer et al., 2018; World Health Organization [WHO], 2021). Fetal Alcohol Spectrum Disorders (FASD) refer to a range of diagnoses following PAE. Recent estimates of the collective prevalence of FASD suggest even higher rates than historically reported at 3.1–9.9% in the United States (May et al., 2018). A population-based study conducted in South Africa found a prevalence of Fetal Alcohol Syndrome (FAS), one of 4 diagnoses under the FASD umbrella term, to be between 5.9–9.1%, and a collective FASD prevalence between 13.5–20.7% (May et al., 2013).

Prenatal tobacco exposure (PTE) is a common co-occurring exposure with PAE (Cornelius and Day, 2009), and has been associated with alterations in speech processing, attention, internalizing and externalizing behavior, and brain development (Cornelius and Day, 2009; El Marroun et al., 2014). Despite our understanding of the teratogenic effect of these substances, PAE in conjunction with PTE continue to occur in substance-using societies and pose significant public health challenges.

Existing literature has attributed brain alterations primarily to the teratogenic effect of PAE, with limited consistent examination of other key and often upstream factors that may also shape brain structural development. Likelihood of prenatal substance exposure is closely associated with availability of socioeconomic resources at individual and neighborhood levels (Karriker-Jaffe, 2013; Coleman-Cowger et al., 2017). In general, socioeconomic resources, PAE and PTE are associated with hardships in prenatal and postnatal experiences, which can also alter a child’s developmental trajectory (Gibson et al., 2009; Lange et al., 2013; Baglivio et al., 2017; Breen et al., 2018; Kambeitz et al., 2019; Luby et al., 2019). Limited recent research provides initial evidence that PAE may interact with low socioeconomic resources (Coles et al., 2019; Uban et al., 2020) to impact child developmental outcomes. With such limited knowledge, more understanding of upstream factors that contribute to teratogenic outcomes on childhood-adolescent brain outcomes is needed.

Existing literature demonstrates that lack of socioeconomic resources is a childhood adversity on its own that leads to disadvantages in executive functioning, memory, and language development (Noble et al., 2006, 2012; Noble and Farah, 2013), and is reflected in development of brain structure (Gonzalez et al., 2020). The Adverse Childhood Experiences (ACEs) framework incorporates factors such as emotional and physical abuse, domestic violence toward the mother, household substance use and mental illness, and household member with a history of incarceration. Although conceptually limited access to socioeconomic resources may be an ACE in and of itself, socioeconomic resources and ACEs have distinct differences. ACEs have been shown to be associated with greater risk for health challenges in children, including risk for mental health challenges, development of chronic medical conditions, and regional brain development alterations (Teicher et al., 2012, 2016; Kerker et al., 2015; Luby et al., 2019; Mall et al., 2020; Sevenoaks et al., 2022).

Less is known about how poverty may increase the likelihood of other ACEs (Melchior et al., 2007; Finkelhor et al., 2013). The conceptual model developed by Culhane and Elo (2005) hypothesized that socioeconomic resources can influence either positive or negative individual health behaviors (including substance use during pregnancy), through the availability of social services, exposure to stress, and social norms. These individual health behaviors may partially explain the association between socioeconomic resources, childhood subsequent ACEs, and child neurodevelopmental outcomes. In other words, low socioeconomic resources, presence of PAE and PTE and more ACEs may tend to cluster together, while each has its own impact on child neurodevelopment.

Some support for the importance of considering socioeconomic resources and ACEs in PAE exists within samples including biological birthing parents. It is established in perinatal literature that socioeconomic resources are associated with PAE, partly via differential patterns and profiles of co-exposures. Lower maternal income is associated with a higher odds ratio of prenatal exposure to marijuana and tobacco (Coleman-Cowger et al., 2017). Women with residence in disadvantaged neighborhoods were more likely to experience substance exposed pregnancies to tobacco and other drugs in comparison to women living in middle-class neighborhoods (Karriker-Jaffe, 2013). Social capital of the country in which women resided was significantly associated with PTE (Shoff and Yang, 2013). Levels of neighborhood assistance accounted for significant variances of type of PAE and PTE after controlling for individual-level characteristics such as race, age, public assistance, and prenatal care (Finch et al., 2001). The potential bidirectionality between upstream socioeconomic resources factors of prevalence of PAE/PTE is not understood well.

In addition to systemic factors, prenatal substance exposure status may serve as indicators of other adverse circumstances within the home environment that shape children’s living experiences. For instance, alcohol use for women has been associated with higher risk for experiencing intimate partner violence, which may be associated with an unstable household environment for the children (O’Connor et al., 2006). Literature shows that maternal ACEs is associated with increased risk of PTE as well as adverse experiences of offspring, such as intimate partner violence and child maltreatment (Pear et al., 2017; Buffarini et al., 2022). It is possible that maternal cumulative exposure to adversity, including ACEs and poverty, increases the risk of prenatal tobacco exposure, which links to a subsequent elevated ACEs in children.

Expanding upon current understanding of how socioeconomic resources and ACEs contribute to PAE- and PTE-related structural brain alterations, we applied a novel conceptual model in the present analyses to examine PAE and PTE as mediators of socioeconomic resources and postnatal ACEs, and to examine the effects of socioeconomic resources, PAE/PTE, and ACEs on brain outcomes. Rather than framing prenatal substance exposure as primary predictors of brain alterations, this intentional reframing of prenatal substance exposure as a mediator is warranted, given the commonly co-occurring of between prenatal substance, socioeconomic resources (Bingol et al., 1987; McLachlan et al., 2020) and ACEs (Kambeitz et al., 2019; Andre et al., 2020): all factors known to individually impact brain development (Rivkin et al., 2008; Dannlowski et al., 2012; Noble et al., 2012; Luby et al., 2013; Bick and Nelson, 2016).

**FIGURE 1:**
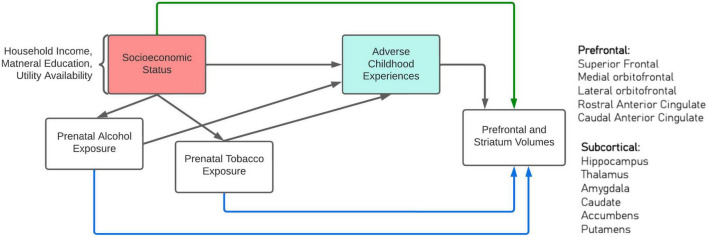
Conceptual model.

For the first aim, we hypothesized that fewer socioeconomic resources would be associated with more ACEs, and presence or absence of PAE or PTE would partially mediate this relationship (Figure 1). The second aim examined whether socioeconomic resources-related resources, PAE or PTE, or ACEs altered cortical brain structural development among children and adolescents (Figure 1). We hypothesized that lower socioeconomic resources, the presence of PAE or PTE, and higher ACEs would be associated with smaller cortical volumes.

## 2. Materials and methods

### 2.1. Study design and participant recruitment

The current study involved a subsample of the existing birth cohort of the Prenatal Alcohol in Sudden Infant Death Syndrome (SIDs) and Stillbirth (PASS) Network recruited from Cape Town, South Africa (Dukes et al., 2014). For the original PASS cohort, pregnant women were recruited during their routine antenatal care at the Belhar antenatal clinic and Bishop Lavis Midwife Obstetric Unit between August 2007 and January 2015. Enrollment of pregnant women started between the 6th week of gestation and delivery day. Pregnant women within this cohort originated from Bishop Lavis and Belhar communities: both low-income urban suburbs that developed as a direct result of apartheid in of Cape Town, South Africa. Historically, both communities have experienced high rates of prenatal alcohol exposure, SIDS and socioeconomic inequalities (May et al., 2000). A detailed report on the recruitment methodology of the original PASS study has been published elsewhere (Dukes et al., 2014).

For the study reported here, birth parent/legal guardian and their child were recruited from the PASS birth cohort among those with surviving children 8–12 years later. This age range was selected for the dynamic pubertal maturation that occurs during the transition from childhood to early adolescence. This period was hypothesized to be more opportunistic for observing lasting brain alternations following prenatal conditions. Further, the neuroimaging protocols were adapted from the Adolescent Brain and Cognitive Development Study, designed for collecting MRI data at this age range. Female birth parents and their children were approached for neuroimaging and other neuropsychological measures in the townships around Cape Town, South Africa. This current analysis includes a sample of 313 birth parent/legal guardian–child participant dyads. The demographics of the study sample is presented in Table 1.

**TABLE 1:** Demographic information.

	NoAlc (N = 98)	EarlyAlc (N = 58)	ContinuedAlc (N = 113)	Total (N = 269)	p-value
PTE					<0.001
NoTob	55 (56.1%)	23 (39.7%)	32 (28.3%)	110 (40.9%)	
Tob	43 (43.9%)	35 (60.3%)	81 (71.7%)	159 (59.1%)	
Sex					0.403
Male	53 (54.1%)	27 (46.6%)	51 (45.1%)	131 (48.7%)	
Female	45 (45.9%)	31 (53.4%)	62 (54.9%)	138 (51.3%)	
Age in years					0.988
Mean (SD)	9.92 (1.27)	9.90 (1.33)	9.93 (1.28)	9.92 (1.28)	
Range	8.00–12.00	8.00–12.00	8.00–12.00	8.00–12.00	
Electricity					0.231
No	0 (0.0%)	0 (0.0%)	2 (2.0%)	2 (0.8%)	
Yes	95 (100.0%)	49 (100.0%)	97 (98.0%)	241 (99.2%)	
Phone					0.022
No	7 (7.4%)	9 (18.4%)	21 (21.2%)	37 (15.2%)	
Yes	88 (92.6%)	40 (81.6%)	78 (78.8%)	206 (84.8%)	
Water					0.612
No	17 (17.9%)	9 (18.4%)	23 (23.2%)	49 (20.2%)	
Yes	78 (82.1%)	40 (81.6%)	76 (76.8%)	194 (79.8%)	
Toilet					0.387
No	31 (32.6%)	21 (42.9%)	40 (40.4%)	92 (37.9%)	
Yes	64 (67.4%)	28 (57.1%)	59 (59.6%)	151 (62.1%)	
Maternal education					0.077
Mean (SD)	10.03 (1.76)	10.38 (1.46)	9.73 (1.69)	9.98 (1.68)	
Range	5.00–13.00	7.00–13.00	4.00–13.00	4.00–13.00	
Household income in South African Rand (ZAR)					0.023
Mean (SD)	932.98 (590.83)	856.47 (617.28)	689.77 (443.20)	817.12 (547.94)	
Range	142.86–3000.00	100.00–3000.00	50.00–1666.67	50.00–3000.00	
ACE total score					0.003
Mean (SD)	3.39 (2.14)	3.36 (2.61)	4.37 (2.30)	3.80 (2.36)	
Range	0.00–10.00	0.00–12.00	0.00–10.00	0.00–12.00	
Parent-reported pubertal development					0.705
Pre	54 (71.1%)	27 (61.4%)	62 (68.1%)	143 (67.8%)	
Early	14 (18.4%)	9 (20.5%)	18 (19.8%)	41 (19.4%)	
Mid	7 (9.2%)	5 (11.4%)	9 (9.9%)	21 (10.0%)	
Late	1 (1.3%)	3 (6.8%)	2 (2.2%)	6 (2.8%)	
Child-reported pubertal development					0.352
Pre	60 (69.8%)	26 (59.1%)	63 (65.6%)	149 (65.9%)	
Early	18 (20.9%)	9 (20.5%)	20 (20.8%)	47 (20.8%)	
Mid	4 (4.7%)	8 (18.2%)	9 (9.4%)	21 (9.3%)	
Late	4 (4.7%)	1 (2.3%)	4 (4.2%)	9 (4.0%)	
Birth weight					0.446
Mean (SD)	3031.11 (500.18)	2955.40 (413.57)	2948.57 (495.20)	2981.97 (481.42)	
Range	1120.00–4905.00	2000.00–3940.00	1400.00–4200.00	1120.00–4905.00	
Prenatal Meth					0.041
Yes	96 (97.0%)	47 (87.0%)	94 (94.9%)	237 (94.0%)	
No	3 (3.0%)	7 (13.0%)	5 (5.1%)	15 (6.0%)	
Prenatal marijuana					0.256
Yes	94 (94.9%)	48 (88.9%)	89 (89.0%)	231 (91.3%)	
No	5 (5.1%)	6 (11.1%)	11 (11.0%)	22 (8.7%)	

Classification of pubertal development follows Cheng et al. (2021).

Inclusion criteria for the birth parents were (1) at least 16 years of age and (2) spoke either English or Afrikaans. Children were between 8 and 11 years of age at the acquisition of the MRI brain scan. Exclusion criteria were (1) history of traumatic brain injury, (2) presence of major medical or central nervous system disorders, and (3) MRI contraindications, such as orthodontic braces and ferromagnetic metal implants.

### 2.2. Measurements

#### 2.2.1. Structural magnetic resonance imaging data acquisition

A 3-Tesla Siemens Skyra scanner at the Cape Universities Imaging Center (CUBIC) was used to acquire whole-brain T1-weighted images for all participants. The total acquisition time was around 45 min, and only data from the structural scan was analyzed for the current study. The image was acquired through a multi-echo T_1w_ MPRAGE sequence, with acquisition parameters as following: 1 × 1 × 1 mm voxel size, 176 slices, slice thickness 1.00 mm, FOV 256 × 256, TR = 2,530 ms, TE = (1.61; 3.44, 5.27; 7.1 ms), TI = 1,240 ms, flip angle = 7 degrees.

#### 2.2.2. Image processing

FreeSurfer’S v5.3 recon-all pipeline was utilized as metrics for volumetric segmentation. Briefly, the FreeSurfer pipeline includes motion correction (Reuter et al., 2010), non-uniform intensity normalization (Sled et al., 1998), skull-strip (Ségonne et al., 2004), Talairach transformation and volumetric labeling of cortical and subcortical regions (Fischl et al., 2002; Fischl, 2004), tessellation of gray/white matter boundaries for topology correction and cortical surface construction (Fischl et al., 2002; Fischl, 2004), parcellation of white and gray matter and derivation of cortical and subcortical matrices. A detailed description of all steps can be found elsewhere: https://surfer.nmr.mgh.harvard.edu/fswiki/FreeSurferMethodsCitation. The structural MRI sequence was adapted from the US-based ABCD Study © that was designed to optimize pediatric neuroimaging for similar age ranges (9.0–10.99 years old): covering both late childhood and early adolescence matching the age range and pubertal maturation of participants in the present study.

#### 2.2.3. ROIs

The overlapping cortical and subcortical regions that have been historically shown to be impacted by PAE and PTE, socioeconomic resources, and ACEs were selected as Regions of interests (ROIs) (Cortical: superior frontal, medial and lateral orbitofrontal, rostral, and caudal anterior cingulate regions; Subcortical: hippocampus, thalamus, amygdala, caudate, nucleus accumbens, and putamen). Volumes of ROIs were analyzed bilaterally across left and right hemispheres.

#### 2.2.4. Socioeconomic resources measures

Socioeconomic resources measures included monthly household income in South African rand (ZAR), the number of school grades completed by the birth parent, dichotomous (yes/no) utility variables that recorded the availability of electricity, phone (landline and/or mobile phone), flushing toilet, and running water in the household (Myer et al., 2008). Socioeconomic resources measures were included individually in the analysis (e.g., household income, utility availability, and maternal education).

#### 2.2.5. Prenatal substance exposure measures

The PASS study collected prospective information on PAE and PTE using a modified Timeline Follow-Back (TLFB) during pregnancy. The TLFB measure was modified to be administered in the participant’s language of choice (Afrikaans in the current analytical sample), and prompts to the researchers were inserted to facilitate precision of administration among participants and across repeated time points within participants during pregnancy (Dukes et al., 2014).

Data on PAE and PTE was collected up to three times during pregnancy (20–24, 28–32, and 34+ gestational weeks) and 1 month post-delivery using the TLFB. Detailed information to accurately measure the total grams of alcohol consumed on a drinking day were collected. Standard drinks were calculated based on the type of alcohol consumed, whether the drinks contained ice, if drinks were shared amongst others, and the volume potentially consumed as measured by the size of container.

Timing data for PAE was grouped into three PAE categories: (1) early PAE, (2) extended PAE, and (3) no PAE. The no PAE group included children whose birth mothers reported consuming no alcohol in all three trimesters. The early PAE group included children whose mother reported having one or more drinks during the first trimester (<12 weeks into pregnancy) but not in the second or the third trimesters, while the extended PAE group included children whose mother reported consuming one or more drinks in two or all trimesters of their pregnancy. Available PTE data was grouped dichotomously into (1) PTE at any time *in utero* or (2) no PTE exposure throughout *in utero* development.

#### 2.2.6. ACEs measure

Because no prior ACE questionnaires existed that were validated for youth in the Cape Town Flats, validated ACEs items from existing literature were compiled across several questionnaires. Individual ACE items were selected in close consultation with research staff in Cape Town to determine which items were: (1) relevant to the lived experiences of the youth participants in their culture; (2) did not require mandatory reporting if endorsed to avoid harming rapport between researchers and the community members within the Cape Town Flats; and (3) retained original meaning after being translated and back-translated into Afrikaans, as determined by the US and South African researchers.

The final ACE questionnaire consisted of 14 dichotomous questions (Supplementary Table 1). The children were asked questions which related to whether they had witnessed sexual abuse, or had experienced emotional and physical, neglect and parental separation, substance use, incarceration and mental illness within the household, homelessness or violence, and loss of a loved one. Child participants completed the questionnaire with the research assistant in their preferred language of either English or Afrikaans. A summary score was calculated by counting the total number of questions that the child endorsed.

### 2.3. Statistical analysis

CRAN R v.4.1 was used to perform statistical analyses (Bates et al., 2015; Kuznetsova et al., 2017; R Development Core Team, 2019; Wickham et al., 2019, 2022; Heinzen et al., 2021).

#### 2.3.1. Mediation analysis

To test whether PAE or PTE were mediators between socioeconomic resources and ACEs, we applied the Baron and Kenny criteria for mediation analysis (Baron and Kenny, 1986). The analytic flow is shown in Figure 1. For the first step, we examined the association between socioeconomic resources variables and ACE total score. A generalized linear model (GLM) was fitted with ACE total score as the outcome variable and household income, maternal education, phone, water, and electricity availability as the explanatory variable with a link function for the Gaussian distribution. For the second step, we tested the association between the explanatory variable and the mediator. A GLM was fitted with the same socioeconomic resources variables as the explanatory variables, and PAE or PTE as the outcome variable, with a link function for the binomial distribution. If PAE or PTE was significantly associated with socioeconomic resources variables, it was then included in the last step of the analysis. For the third and last step of the mediation analysis, we examined the direct effect between socioeconomic resources and ACE total score by adjusting for the potential mediator. The condition for a partial mediation was met if (1) the main outcome variable, ACE total score, and the mediator, PAE or PTE, were significantly associated with socioeconomic resources variables; (2) the mediator was significant in the third step analysis; (3) the absolute values of the estimate of the explanatory variables were reduced when the mediator was included.

#### 2.3.2. sMRI analysis

To examine whether PAE/PTE, socioeconomic resources, or ACE were associated with brain volume alterations in the prefrontal and striatum areas, we applied a linear mixed-effects model using the lme4 package in R (Bates et al., 2015; Kuznetsova et al., 2017). Because PAE/PTE, socioeconomic resources and ACE were significantly associated with each other, we examined the effect of PAE/PTE, socioeconomic resources, and ACE on the ROI volumes separately. Hemisphere was included as a within-subject variable. Our models were constructed as follows: first, we constructed a reduced model with only the primary relationship; then, we built up from the reduced model by adjusting for covariates, including child age and sex. In the case of PAE and PTE, we constructed a third model with an interaction term between PTE and PAE and the covariates. We used AIC comparison and log likelihood ratio test to determine whether including the covariates provided a better fit to the model and whether an interaction was appropriate. Lastly, a false discovery rate (FDR) correction was applied to all individual explanatory variables across the 11 ROIs. The results were considered significant if the *q*-value, the FDR analog of the *p*-value, was less than 0.05.

### 2.4. Ethics

The data collection was approved by the Human Research Ethics Committee of the Faculty of Health Sciences of University of Cape Town (HREC UCT REF 248/2014). The Human Research Ethics Committee of the Faculty of Health Sciences of Stellenbosch University gave their ethical approval (REF 248/2014). The Institutional Review Board (IRB) at Children’s Hospital in Los Angeles approved the processing of de-identified neuroimaging data (CHLA-19-00228). The IRB at University of California, Irvine approved the analysis of de-identified data (UCI #212354).

## 3. Results

Detailed demographic information is presented in Table 1. Of the 313 enrollees, 229 participants (mean age: 9.91 years; 131 (48.7%) male) had available PAE and PTE data. Among them, 50 had early PAE (exposure during the 1st trimester), 100 had extended PAE, and 95 had no PAE. A 110 had no PTE and 159 had PTE. Fifty-five had only PAE, 43 had only PTE, and 116 had both PAE (early or extended) and PTE. On average, the total number of ACEs endorsed was 3.8. The average maternal education was 9.98 years, while the mean monthly household income was 817.12 ZAR (equivalent to $45.55 US dollars). Age, sex, parent-reported and child-reported pubertal development scale did not differ by PAE status (*p* > 0.05). Birth weight also did not differ by PTE and PAE (*p* > 0.05).

### 3.1. Mediation analysis

In the first-step mediation analysis, we examined the primary relationship between specific socioeconomic resources-related resources and total ACE scores. Lower maternal education (β = −0.293,*p* = 0.01) and no phone access (β = −0.968,*p* = 0.05) were both associated with higher ACE total scores. For the second-step analysis, socioeconomic resources was regressed against the two potential mediators, PAE and PTE. Lower household income (β = −0.001,*p* = 0.01) and lower maternal education (β = −0.248,*p* = 0.05) were associated with PTE, while no phone access only (β = −1.210,*p* = 0.05) was associated with PAE (i.e., early PAE, extended PAE, no PAE). In the third step of mediation analysis, PTE (i.e., yes PTE, no PTE) and PAE were included, respectively in the primary association models to test the direct association between socioeconomic resources and ACE after adjusting for the mediators. PAE was not a significant explanatory variable when the model included socioeconomic resources variables, and therefore PAE did not fulfill the criteria as a mediator. PTE remained significant when added to the socioeconomic resources-ACE model (β = 1.110,*p* = 0.01), where the presence of PTE was associated with higher ACE total scores. Moreover, the absolute value of the effect estimates of household income and maternal education were reduced after PTE was included in the model (Table 2). Therefore, PTE fulfilled the criteria as a partial mediator between socioeconomic resources and ACE, while PAE did not fulfill the criteria as a partial mediator.

**TABLE 2:** Coefficients and 95% confidence intervals of mediation models.

	ACE total score		PTE	PAE
	1	2	3	4
Monthly household income	−0.001	−0.0004	−0.001**	−0.001
	(−0.001, 0.00001)	(−0.001, 0.0002)	(−0.002, −0.0003)	(−0.001, 0.00003)
Maternal education	−0.293**	−0.235*	−0.248*	0.085
	(−0.492, −0.093)	(−0.434, −0.037)	(−0.449, −0.047)	(−0.100, 0.270)
Phone access	−0.968*	−0.852	−0.523	−1.213*
	(−1.913, −0.023)	(−1.778, 0.074)	(−1.487, 0.441)	(−2.270, −0.156)
Water access	0.174	0.304	−0.581	−0.062
	(−0.797, 1.144)	(−0.648, 1.255)	(−1.539, 0.377)	(−0.995, 0.870)
Toilet access	−0.218	−0.274	0.271	−0.39
	(−1.051, 0.615)	(−1.089, 0.540)	(−0.533, 1.075)	(−1.175, 0.396)
Prenatal tobacco exposure		1.108**		
		(0.403, 1.814)		
Akaike inf. crit.	826.371	818.773	234.663	242.572

*p < 0.05, **p < 0.01.

### 3.2. sMRI analysis

**FIGURE 2:**
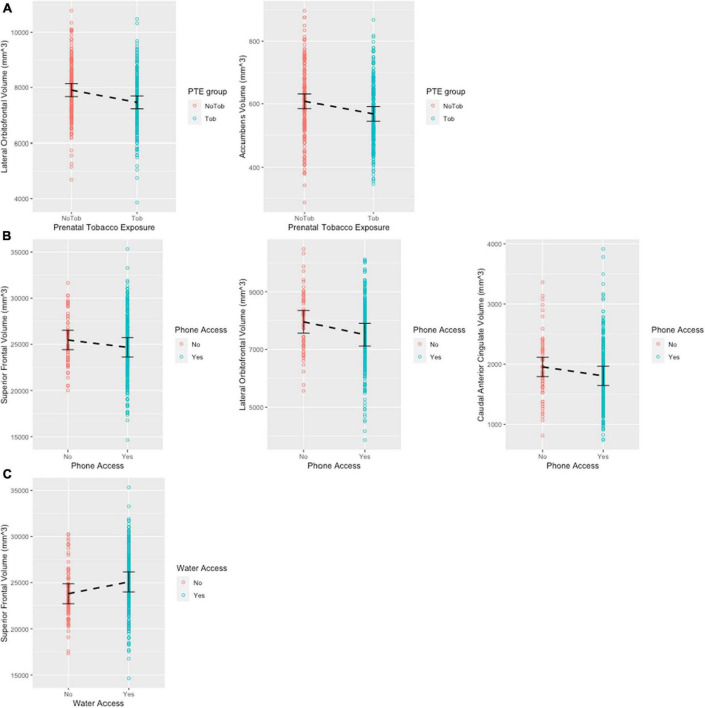
Scatter plots showing significant regions post FDR correction. Error bars show 95% confidence interval. **(A)** Significant associations between prenatal tobacco exposure and brain volume. **(B)** Significant associations between phone access and brain volume. **(C)** Significant associations between water access and brain volume.

All models were adjusted for age (months) and biological sex (at birth), as the covariates significantly improved model fit as evident in log likelihood ratio tests (*p* < 0.05). The PAE and PTE models did not include the interaction term between PTE and PAE, as in all cases the interaction term did not significantly improve model fit. After FDR correction, PTE was significantly associated with the lower volumes of lateral orbitofrontal region (β = −331.000, *q* = 0.033) and accumbens areas (β = −34.800, *q* = 0.033) (Figure 2A). PAE was associated with increased thalamus, accumbens area and caudate before the FDR correction (*p* < 0.05), but these associations did not carry on with the FDR correction (*q* > 0.05). ACE total score was not significantly associated with the volumes of any of the 11 brain ROIs. Phone access was associated with the smaller volumes of superior frontal (β = −1336.036, *q* = 0.046), lateral orbitofrontal (β = −513.865, *q* = 0.046), and the caudal anterior cingulate (β = −222.982, *q* = 0.046) (Figure 2B). Water access was associated with larger volumes of the superior frontal region (β=1569.527, *q* = 0.013) (Figure 2C). Uncorrected *p*-values and full model estimates are presented in Supplementary Table 2.

## 4. Discussion

The present analyses examined a novel reframing of PAE and PTE as mediators for the association between socioeconomic resources and postnatal ACEs on cortical brain volumes. Within a very low socioeconomic resources context, and with prospective community-levels of prenatal substance exposure, we found that PTE, but not PAE, partially mediated the association between less *in utero* socioeconomic resources and subsequent more postnatal ACEs for the youth. Lower socioeconomic resources during pregnancy were associated with higher likelihood of PTE, and in turn PTE was associated with higher total number of endorsed ACEs. Both socioeconomic resources and PTE were associated with smaller volumes in prefrontal and striatum regions. Lower socioeconomic resources during pregnancy were associated with increased likelihood of subsequent PAE. However, given null brain findings with community-levels of PAE in this cohort, whether PAE plays a similar mediating role between socioeconomic resources, ACEs and brain outcomes as PTE does remains unknown, particularly for populations experiencing higher PAE known to cause clinical FASD, or within higher resourced contexts.

Our results demonstrate that PTE, but not PAE, was associated with lower cortical volume in the lateral orbitofrontal region and nucleus accumbens. While lateral orbitofrontal has been consistently associated with processing of rewards and punishments, as well as emotional and social regulations (Kringelbach and Rolls, 2004), nucleus accumbens serves to integrate information from frontal and temporal regions and facilitate action (Floresco, 2015). These functional correlates of lateral orbitofrontal cortex and nucleus accumbens are consistent with the negative association between PTE and global cognition in children between 9 to 12 years old found in the current literature, suggesting a potential brain-behavior relationship (Fried et al., 1998; Gonzalez et al., 2023). Our results are consistent with prior studies from this same birth cohort representing community patterns of PAE and PTE, showing more widespread cortical and subcortical brain alterations with PTE compared to PAE at ages 6 years old (Uban et al., 2023) and ages 8–12 years (Marshall et al., 2022). Compared to alcohol, tobacco use is less likely to be cut back during pregnancy and more likely to be associated with tobacco exposure after pregnancy (Leech et al., 1999; Cornelius and Day, 2000). Even among women who reduce their tobacco use or quit spontaneously during pregnancy, postpartum relapse is common (Crume, 2019). It is likely that children who had PTE were also exposed to prolonged second-hand smoke perinatally [from birth parent and/or others smoking around child (Paavola et al., 1996; Scherrer et al., 2012)], which has been known to increase the risk of poorer neurodevelopmental outcomes in children (Chen et al., 2013). Therefore, it is possible that the structural brain development differences observed in our analysis were the result of accumulated tobacco exposure via maternal systems from both maternal use as well as use from others via second-hand smoke exposure during perinatal development. The potential for PTE from others’ use may be a mechanism for reaching higher doses of exposure, and/or longer durations of exposure across postnatal developmental stages, unlike PAE. Together, these mechanisms for PTE that are unique from those of PAE may explain the more widespread effects on brain development at ages 6 (Uban et al., 2023) and 8–11 years as seen here and in Marshall et al. (2022). Interestingly, PTE dose-response relationships did not show significant results after corrections for multiple comparisons (Marshall et al., 2022), suggesting that PTE exposure from the postnatal period, or relating to perinatal tobacco exposure from others around the pregnant person or baby may be driving PTE outcomes more than maternal use in pregnancy alone. Data on the existence of postnatal tobacco exposure was not collected in our sample, which limited our ability to disentangle prenatal from postnatal tobacco exposure. Future research may investigate whether there is a dose-response relationship between prenatal and postnatal tobacco exposure and structural brain development.

While we are aware of the teratogenic effects of exposure to substances *in utero*, there exist other mechanistic pathways by way of hypoxia that might affect brain development, including the presence of obstetric complications. Obstetric complications, including preeclampsia, eclampsia, and gestational diabetes, can affect brain volumes (Rätsep et al., 2016; Luo et al., 2022). Our analyses are potentially limited by not accounting for obstetric complications that may affect brain morphology *in utero* with continuing effects seen in the growth trajectory of the developing brain through adolescence. Additionally, PTE has been shown to be associated with lower birth weight, smaller head circumference, and shorter length in newborns (Cornelius and Day, 2000). Specifically in our sample, birth weight did not differ by PTE or PAE status, again suggesting that prospective data most likely reflects community-level patterns of exposure and not necessarily high doses that are commonly seen in clinical FASD research samples.

Our analysis showed that phone access (landline and/or mobile phone) and running water access *in utero* were associated with volumes of the frontal regions in our sample of children between 8 to 11 years old in Cape Town, South Africa. Most of the existing literature on the impact of socioeconomic resources on child brain development have included samples from United States. How socioeconomic resources influence child development may be substantially different in a community where access to basic needs is inconsistent. Phone access is not universal in Cape Town, South Africa, because the necessary hardware to support phone service is expensive due to importation and little domestic manufacturing, and cellular data prices are exorbitantly high for lower resourced communities (Walton and Donner, 2012). Running water access for the Cape Town participants in this study is also negatively impacted by the legacy of racial inequality, where restricted access to clean and consistent water supply remains common (Enqvist and Ziervogel, 2019). Therefore, phone and water are likely proxies of the physical environment, such as access to governmental supports for maintaining utilities or exposure to environmental toxins or nutrition. Together, these physical environmental factors may impact child brain development and are potentially associated with housing amenity-based factors impacting ventilation of cooking, sanitation, and neighborhood safety. Given that our sample was derived from a low-resource community, it is also possible that the associations between utility access and structural brain volume may not generalize to communities in developed countries with more resources and infrastructure. Additionally, access to socioeconomic resources has been intertwined with cross-generational race/ethnicity-based oppression. Thus, the brain alterations we found as a function of socioeconomic resources in this sample may also reflect the impact of experienced racism, in addition to environmental exposures and poverty. Further research is needed to assess the interaction between racism, environmental exposure and poverty, and their collective impact on brain development.

Socioeconomic resources, but not ACEs, were related to lower cortical volumes, and less socioeconomic resources were associated increased likelihood of PTE and PAE. The presence of PAE/PTE may be a symptom of existing socioeconomic inequities, which may continue to independently and/or interactively impact the postnatal experience of the child. It is possible that, in this sample of participants, PTE and PAE are symptoms of less access to resources. Substances, including tobacco and alcohol, are commonly used to cope with stressors, including those relating to additional economic and low resourced living conditions (O’Connor et al., 2011; Peer et al., 2014; Watt et al., 2014). PTE may be reflective of additional needs to cope with stressors in a lower socioeconomic resources context in our study. Indeed, tobacco use among women in low resourced communities around Cape Town has been associated with poverty and more psychosocial stress (Peer et al., 2014). Previously, more adverse life events and a perception of lack of control over one’s environment were found to be associated with an increased risk of tobacco use among this population (Peer et al., 2014). Thus, the present study provides evidence to extend established socioeconomic resources and PTE associations to the period of pregnancy, and subsequent ACEs endorsed by their children. With intentional incorporation of these factors in PAE brain research, more can be understood about the complex interplay between co-occurring contributing factors with PAE/PTE on brain structure development. It is possible that teratogenic potential of PAE/PTE may differ as a function of many factors, including co-occurring exposures, socioeconomic resources, and variable postnatal experiences.

We did not find an association between the total number of ACEs and brain volume in the prefrontal and striatum regions. While the 14-point ACE scoring system captures the grouped experience of the adverse events, the cumulative score is not specific to the three domains of neglect, abuse, and household challenges scored on the ACE scale. Moreover, the scoring of events such as this on a linear scale deprives us of the sensitivity to the chronicity and intensity of the events. The screening of ACEs may not fully capture the breadth of adverse events experienced by children living in post-apartheid South Africa and perhaps better serves as preliminary data on ACEs for the PASS birth cohort. There are cultural differences in how people experience, and express abuse, neglect, and household challenges compared to the U.S. population with whom these ACE items were first developed. We attempted to minimize the cultural effects through forward- and back-translation, but there may be persisted issues of cultural validity with the measure. In addition, children may not remember adverse events that happened when they were very young and therefore might not report these events accurately, if at all. Given that early childhood is an especially sensitive developmental period, the limitation of the child self-report may also have contributed to our lack of findings. ACEs requiring mandatory reporting were not assessed and may have artificially created a ceiling effect on total ACE scores. Lastly, resilience is known to be important as an interacting force to ACEs and warrants further investigation to understand how it relates to PAE and/or PTE, socioeconomic resources and ACEs for these brain outcomes.

Although not directly tested, known mechanisms implicate stress systems for underlying, in part, the impact of socioeconomic resources and ACEs on brain development. The toxic stress model hypothesizes that poverty and maltreatment influence levels of adversity, which contribute to toxic stress and allostatic load and thus affect brain and cognitive development (McEwen and McEwen, 2017). In this model, toxic stress activates the hypothalamic-pituitary-adrenal (HPA) axis and thereby alters brain structures involved in neuroendocrine functioning, such as the limbic system and the prefrontal cortex (McEwen and McEwen, 2017). Indeed, a substantial body of literature has demonstrated associations between child maltreatment and altered structural and functional connectivity of the fronto-limbic regions (Hanson et al., 2010; Teicher et al., 2012; McLaughlin et al., 2014, 2016; Herzberg and Gunnar, 2020). Similar HPA and brain alterations have been found to associate with PAE. Animal models of implicate HPA-dysregulation as a key mechanism of lasting harm of PAE on brain structural alterations in prefrontal and the limbic regions (Uban et al., 2010, 2013). Few studies to date have examined PAE, PTE, socioeconomic resources, and HPA-function and warrant future investigation.

Additional contextual characteristics of the present study should be noted. Firstly, in the US, the majority of participants in historical PAE brain literature have been recruited from clinical FASD populations and almost always raised as adoptees, outside of their racial/ethnic/culture of origin (Uban et al., 2020). Here, the birth cohort from South Africa was comprised of child and adolescent participants raised by the biological mother, effectively eliminating cultural mismatch or not being raised by the biological mother as drivers of brain alterations observed with PAE. Second, given the prospective nature, PAE and PTE patterns reflected community-level patterns of PAE. The majority of PAE-focused published work identified participants with established facial dysmorphology or severe patterns of PAE, commonly associated with diagnoses such as fetal alcohol syndrome (FAS) or partial FAS (pFAS) (Coles et al., 2020). Community-patterns of PAE with consideration of PTE may better capture FASD-related diagnoses that have been historically underrepresented in FASD clinical brain research, such as alcohol related neurodevelopmental disorder (ARND).

The birth cohort data leveraged in this study is from a low-resource community in Cape Town, South Africa, which has experienced cross-generational stressors through displacement and race/ethnicity-based oppression through historical apartheid. Specific to the Cape Town Flats region where the study participants reside, the physical environment is limited by lasting infrastructure challenges, in part due to the legacy of Apartheid (Henri and Grunebaum, 2005; O’Connell, 2018). Black communities were displaced from the Cape Town city area and rendered to the peripheral where basic infrastructure is lacking even today (Henri and Grunebaum, 2005; O’Connell, 2018). Systemic race-based oppression spanning generations combined with lack of resources have often led to experiences of toxic stress and substance use (Watt et al., 2014). This community has historically been labeled as having high FASD prevalence in research (Croxford and Viljoen, 1999; May et al., 2000; Olivier et al., 2016). Our study is contextualized with this consideration of poverty and systemic race-based oppression. Future research may further examine specific pathways through which poverty and psychosocial stress during pregnancy, as well as PAE/PTE, become associated with ACEs endorsed by children, and whether interventions and community services may disrupt the intergenerational transmission of adversity in this population.

In summary, our findings support the hypothesis that contextual factors, such as access to socioeconomic resources, may impact brain development through multiple pathways, including a direct pathway through the availability of certain resources and an indirect pathway through increasing the risk of teratogenic exposure (e.g., tobacco). These socioeconomic resources are entangled with cross-generational race/ethnicity-based oppression and poverty stemming from the legacy of the Apartheid. Therefore, our findings may not *necessarily* reflect differences in brain development due solely to poverty. Future studies may conceptualize the teratogen exposure as one factor embedded within a web of contextual factors that also influence brain development. Intentional incorporation of contextual factors that can also drive differences in brain development are needed to expand future teratogenic research, and to help destigmatize birth parents. Understanding varying patterns of PAE and PTE in the context of broader socioeconomic resources influences and their connections with postnatal ACEs can present novel policy-level and community-level interventions. This broader understanding of PAE and PTE outcomes may lead to support and awareness for affected individuals that is consistent with current recommendations to address social determinants of substance use.

## Data availability statement

The original contributions presented in this study are included in this article/Supplementary material, further inquiries can be directed to the corresponding authors.

## Ethics statement

The studies involving human participants were reviewed and approved by the Human Research Ethics Committee of the Faculty of Health Sciences of the University of Cape Town (HREC UCT REF 248/2014), the Human Research Ethics Committee of the Faculty of Health Sciences of Stellenbosch University (REF 248/2014), the Institutional Review Board at Children’s Hospital in Los Angeles (CHLA-19-00228), and the Institutional Review Board at the University of California, Irvine (UCI #212354). Written informed consent to participate in this study was provided by the participants’ legal guardian/next of kin.

## Author contributions

SB, DJ, DS, KN, SJ, HO, and KU acquired the funding. YX, VR, SB, DJ, BS, KD, WC, JI, EK, KN, and KU contributed to the data acquisition and processing. YX, VR, HA, and KU contributed to the statistical analysis. YX wrote the manuscript. VR, SB, DJ, BS, KD, DS, HA, and KU contributed to the manuscript. ERS contributed to the funding and manuscript. All authors contributed to the conceptualization of the manuscript and approved the submitted version.
