# Supplementary material for: Contextualizing the impact of prenatal alcohol and tobacco exposure on neurodevelopment in a South African birth cohort: an analysis from the socioecological perspective
Source: Front Integr Neurosci. 2023 Jul 18;17:1104788. doi: 10.3389/fnint.2023.1104788 (PMC10390790; doi:10.3389/fnint.2023.1104788)
Supplement: Supplementary file 1 [file Table_1.DOCX]

Supplementary Material

# Adverse Childhood Experiences (ACEs) in English and Afrikaans

## Adverse Childhood Experiences (English)

1. Did a parent or other adult in the household often or very often swear at you, insult you, put you down, or humiliate you?
2. Did a parent or other adult in the household often or very often push, grab, slap, or throw something at you? Or, ever hit you so hard that you had marks or were injured?
3. Did you often or very often feel that no one in your family loved you or thought you were important or special? Or, your family didn't look out for each other, feel close to each other, or support each other?
4. Did you often or very often feel that you didn't have enough to eat, had to wear dirty clothes, and had no one to protect you?
5. Were your parents ever separated or divorced?
6. Did you live with anyone who was a problem drinker or alcoholic, or who used street drugs?
7. Was a household member depressed or mentally ill, or did a household member attempt suicide?
8. Did a household member go to prison?
9. Have you witnessed a loved one or close friend being physically abused?
10. Have you witnessed a loved one or close friend being sexually abused?
11. Have you experienced homelessness, or witnessed a loved one or close friend being homeless?
12. Have you been in a severe accident, or witnessed a loved one or close friend in a severe accident?
13. Have you experienced any violence related to crime, or witnessed a loved one or close friend involved in a violent event related to crime?
14. Have you experienced the death of a close loved one or close friend?

## Adverse Childhood Experiences (Afrikaans)

1. Het 'n ouer of ander volwassene in die huishouding gereeld of baie dikwels op jou gevloek, jou beledig, jou geminag of verneder?
2. Het 'n ouer of ander volwassene in die huishouding gereeld of baie dikwels ... jou gestoot, gegryp, geklap of jou gegooi met iets? - of jou ooit so hard geslaan dat jy merke gehad het of beseer was?
3. Het jy dikwels of baie dikwels gevoel dat ... Niemand in jou familie vir jou lief was nie of gedink het dat jy belangrik of spesiaal is nie? - of jou gesin het nie na mekaar omgesien, geheg aan mekaar gevoel of mekaar ondersteun nie?
4. Het jy dikwels of baie dikwels gevoel dat ... Jy het nie genoeg gehad om te eet nie, moes vuil klere dra en niemand gehad om jou te beskerm nie?
5. Was jou ouers ooit vervreemd van mekaar of geskei?
6. Het jy saam met iemand gebly met 'n drankprobleem of 'n alkoholis of iemand wat straatdwelms gebruik het?
7. Was 'n lid van die huisgesin depressief of gediagnoseer met 'n geestesongesteldheid, of het 'n huislid selfmoord probeer pleeg?
8. Het 'n lid van die huisgesin tronk toe gegaan?
9. Het jy gesien hoe 'n geliefde of nabye vriend fisies mishandel is?
10. Het jy gesien hoe 'n geliefde of goeie vriend seksueel mishandel is?
11. Het jy al haweloosheid beleef of ervaar dat 'n geliefde of goeie vriend haweloos was?
12. Was jy in 'n ernstige ongeluk, of het jy ervaar hoe 'n geliefde of goeie vriend in 'n ernstige ongeluk was?
13. Het jy enige geweldsverwante misdaad ervaar, of waargeneem hoe 'n geliefde of nabye vriend betrokke was in 'n gewelddadige gebeurtenis wat verband hou met misdaad?
14. Het jy die dood van 'n geliefde of goeie vriend ervaar?

# Supplementary Figures and Tables


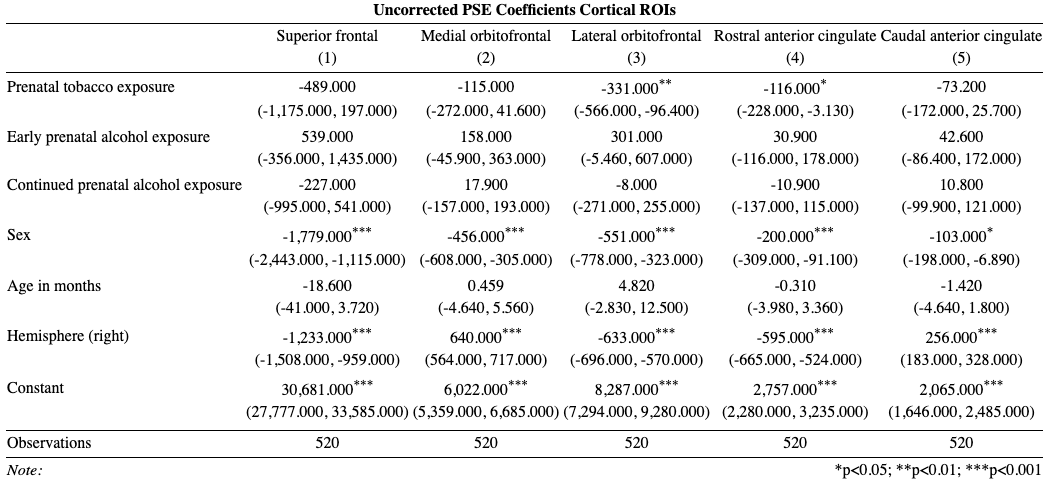

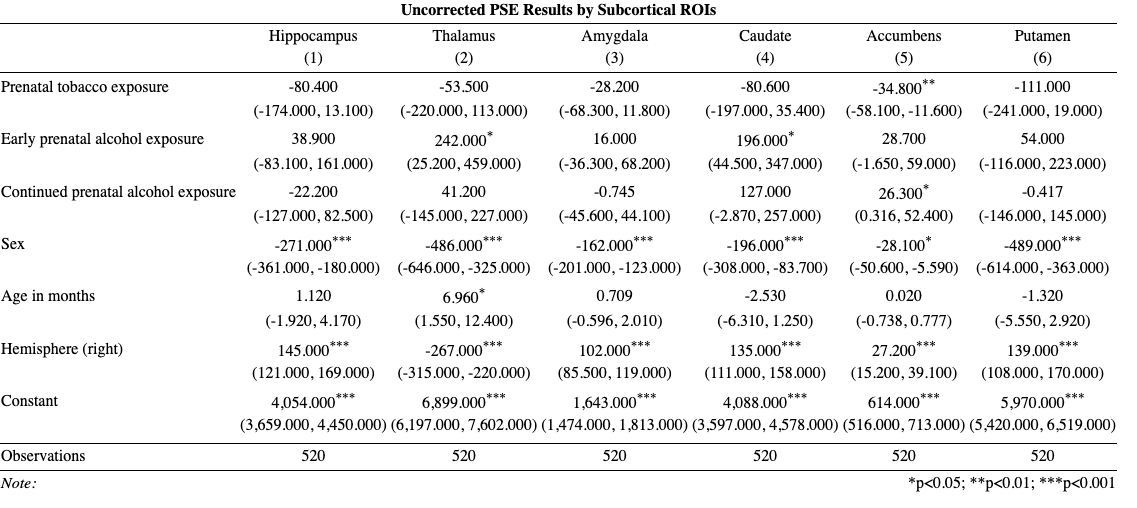


**Supplementary Table 1.** Uncorrected results of PSE on cortical and subcortical ROIs.


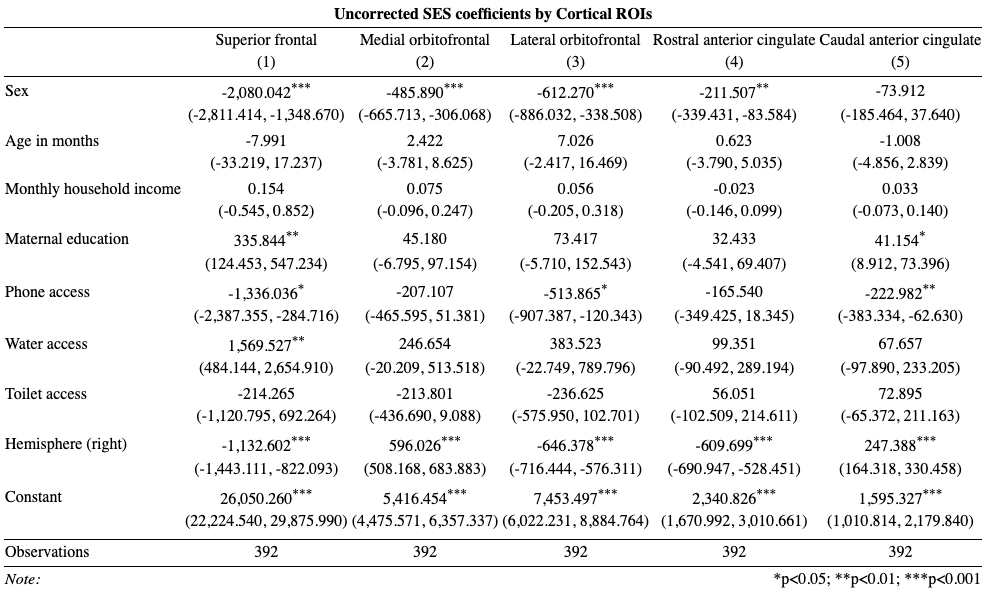


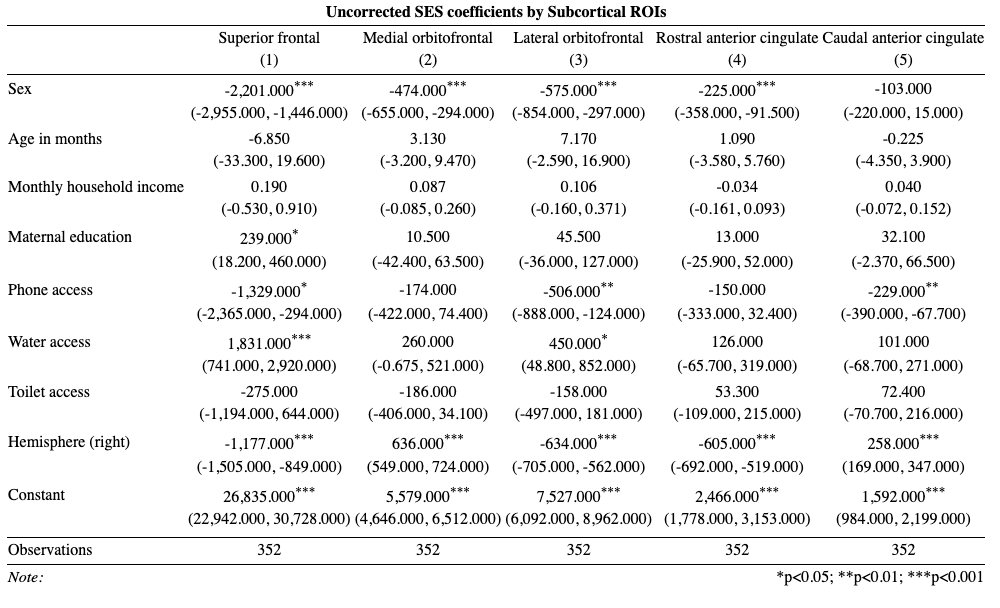


**Supplementary Table 2.** Uncorrected results of SES on cortical and subcortical ROIs.


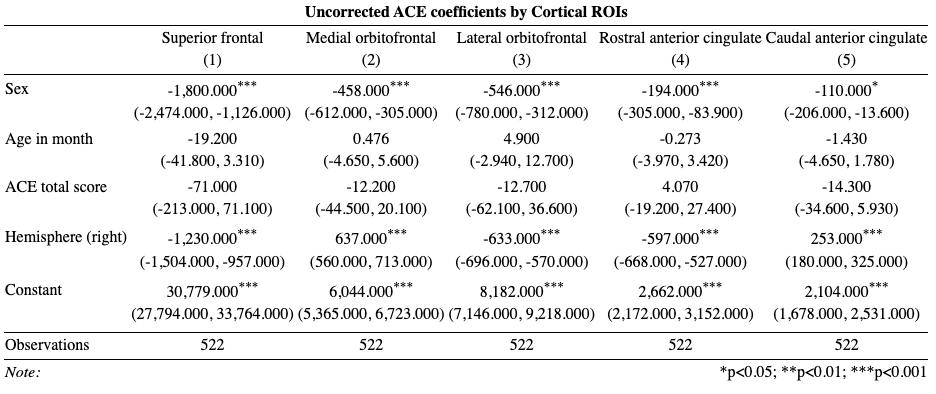


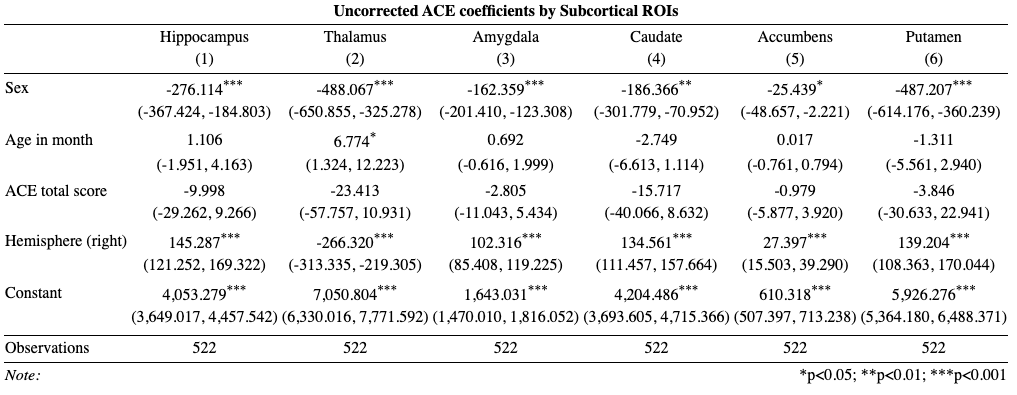


**Supplementary Table 3.** Uncorrected results of ACEs on cortical and subcortical ROIs.
